# Production of Malheuran A, a Geranylated Flavonoid with Antimicrobial and Anti-Inflammatory Activities, in Hairy Root Cultures of *Dalea purpurea*

**DOI:** 10.3390/plants14020259

**Published:** 2025-01-17

**Authors:** Amit Raj Sharma, Gaurav Gajurel, Salma Abdel-Karim, Mohammad Abrar Alam, Robert Colquhoun Shields, Fabricio Medina-Bolivar

**Affiliations:** 1 Arkansas Biosciences Institute, Arkansas State University, Jonesboro, AR 72401, USA; asharma@astate.edu (A.R.S.); gaurav.gajurel@smail.astate.edu (G.G.); salma.abdelkar@smail.astate.edu (S.A.-K.); 2 Molecular Biosciences Graduate Program, Arkansas State University, Jonesboro, AR 72401, USA; 3 Department of Chemistry and Physics, Arkansas State University, Jonesboro, AR 72401, USA; malam@astate.edu; 4 Department of Biological Sciences, Arkansas State University, Jonesboro, AR 72401, USA; rshields@astate.edu

**Keywords:** hairy root cultures, *Dalea purpurea*, elicitation, re-elicitation, malheuran A, geranylated flavonoid, antimicrobial, anti-inflammatory

## Abstract

Phenolic compounds, such as stilbenes and flavonoids, from *Dalea* spp. exhibit diverse biological activities, including antimicrobial, anti-inflammatory, and cytotoxicity properties. To this end, the objectives of this study were to establish hairy root cultures of *D. purpurea* and assess its capacity to produce these bioactive compounds. The co-treatment of the hairy root cultures with the elicitors methyl-β-cyclodextrin, magnesium chloride, methyl jasmonate, and hydrogen peroxide led to the production and secretion of the geranylated flavanone malheuran A, which was confirmed by mass spectrometry and NMR spectroscopy analyses. The highest amount (104.3 ± 6.9 mg/L) of malheuran A was detected at 192 h after elicitor treatment. The elicited hairy roots were re-elicited for 192 h with the same combination of elicitors, and they produced a substantial amount of malheuran A (91.9 ± 6.8 mg/L). Malheuran A, purified from extracts of the hairy root culture medium, showed potent antimicrobial activity against Gram-positive bacteria, including methicillin-resistant *Staphylococcus aureus* and vancomycin-resistant Enterococci. It also demonstrated anti-inflammatory effects by suppressing nitrite production in LPS-stimulated RAW 264.7 macrophages. These findings show that various elicitor treatments can boost malheuran A production in hairy root cultures, making them a sustainable bioproduction platform for this bioactive specialized metabolite.

## 1. Introduction

*Dalea purpurea* Vent., commonly known as purple prairie clover, is a native North American perennial herb. *Dalea* species are prolific producers of phenolic metabolites, with a notable abundance found within the flavonoid class. These flavonoids exhibit diverse biological activities, including antimicrobial, anti-inflammatory, antioxidant, antifungal, and antiparasitic properties. Ethnomedicinal studies have unveiled a rich history of the utilization of *D. purpurea* by Native Americans over centuries. Traditionally, it has been used to treat various ailments, including heart trouble, diarrhea, measles, and pneumonia [1].

The first chemical investigation of *Dalea* species was conducted by Dreyer and co-workers in 1975 on two *Dalea* species, *D. emoryi* and *D. polyadenia*. The compounds coumarin, 5-methoxycoumarin, dalrubone, and methoxydalrubone were isolated from the benzene extract of *D. emoryi*, while *D. polyadenia* yielded 2*S*-demethoxymatteucinol, along with the first three compounds [2]. The Dictionary of Natural Products Database identified 68 hits for *Dalea* species, with only four for *D. purpurea* [3]. A moderate activity of the methanolic extract of *D. purpurea* was observed in an opioid assay. The subsequent fractionation of the methanol extract using silica gel vacuum liquid chromatography led to the isolation of three new geranylated stilbenes, denoted as pawhuskins A–C, and the previously known compound petalostemumol [4]. This suggests that the *Dalea* genus is relatively underexplored, offering the potential for discovering new bioactive natural products.

Plants have a rich historical legacy as sources of bioactive natural products. However, extracting these compounds from field-collected plants poses challenges, such as low yield and seasonal variations in metabolite production [5,6]. To overcome these issues, a plant biotechnological technique, known as hairy root cultures, has been introduced. Hairy root cultures are generated through the infection of plants with *Agrobacterium rhizogenes*. These induced hairy roots exhibit attractive properties, including rapid growth in hormone-free media, chemical, and genetic stability, and the ability to produce a higher quantity of specialized metabolites than the parent plants [7]. Before this research, the establishment and utilization of hairy root cultures of *Dalea* spp. for investigating natural products had not been undertaken. *Dalea* hairy root cultures could be a valuable resource for exploring and producing novel bioactive compounds at a larger scale, opening up exciting prospects for future research and drug discovery.

Flavonoids represent one of the most diverse groups of polyphenols found in plants. Among them, flavanones are a significant subclass that exhibits a wide range of biological activities [8]. Other subclasses of flavonoids are the prenylated, geranylated, and farnesylated flavonoids, characterized by the addition of prenyl (C5), geranyl (C10), and farnesyl (C15) groups to the flavonoid skeleton, respectively. These flavonoid derivatives are relatively rare in nature and are frequently encountered in specific plant families such as Fabaceae and Moraceae, often confined to certain genera such as *Sophora* (Fabaceae), *Morus* (Moraceae), and *Artocarpus* (Moraceae) [9,10]. Notably, *Dalea* flavonoids predominantly contain prenyl (C5) and geranyl (C10) substitutions on the flavonoid backbone [11,12]. This unique characteristic suggests that unexploited species within the *Dalea* genus could potentially serve as a valuable source for discovering novel prenylated and geranylated flavonoids. Furthermore, the low yield from native plants can be overcome by using the hairy root culture of such untapped *Dalea* species with an elicitation technique to obtain large quantities of such rare geranylated flavonoids.

Geranylated flavonoids have recently attracted the attention of researchers due to their diverse biological activities, such as antimicrobial, anti-inflammatory, and cytotoxicity properties [13,14,15]. The activities of geranylated flavonoids may be attributed to the presence of the geranyl side chain along with the phenolic hydroxyl group [16]. The addition of geranyl moieties makes the molecules more active than their parent compounds. This is likely because the geranyl side chain increases the lipophilicity of the molecules and enhances their strong affinity to the biological membrane [17]. Among these activities, the antimicrobial and anti-inflammatory properties of geranylated flavonoids have been extensively studied [13,18,19,20,21,22]. Natural products with antimicrobial and anti-inflammatory effects are highly valuable because they can offer potential alternatives to synthetic drugs, often with fewer side effects and lower toxicity profiles [23].

Antimicrobial resistance poses an imminent threat to public health. Methicillin-resistant *Staphylococcus aureus* (MRSA) and vancomycin-resistant Enterococci (VRE) are among the most encountered human pathogens, raising significant concerns as they are commonly responsible for hospital-acquired infections. Such bacteria resistant to multiple drugs are challenging to treat with conventional antibiotics. Therefore, there is an ongoing search for the discovery of new metabolites to effectively treat drug-resistant microbes [24,25].

Inflammation is a complex biological response to infection or injury and encompasses various mechanisms crucial for the body’s defense. It typically arises when infectious microorganisms like bacteria, viruses, or fungi infiltrate specific tissues or circulate in the bloodstream. Additionally, inflammation may manifest in response to other factors such as tissue injury, cell death, cancer, ischemia, or tissue and organ degeneration [26,27]. Notably, geranylated flavonoids are characterized by both a phenolic component and a terpenoid chain and demonstrate diverse biological activities, showing promising potential as primary candidates for developing anti-inflammatory therapeutic agents [22,28]. Hence, there is always a need to discover new bioactive natural products to treat conditions such as drug-resistant microorganisms and inflammation. Hairy root cultures, coupled with an elicitation approach, could be a sustainable production platform for the enhanced production of bioactive metabolites under controlled conditions to address these ailments.

**Figure 1:**
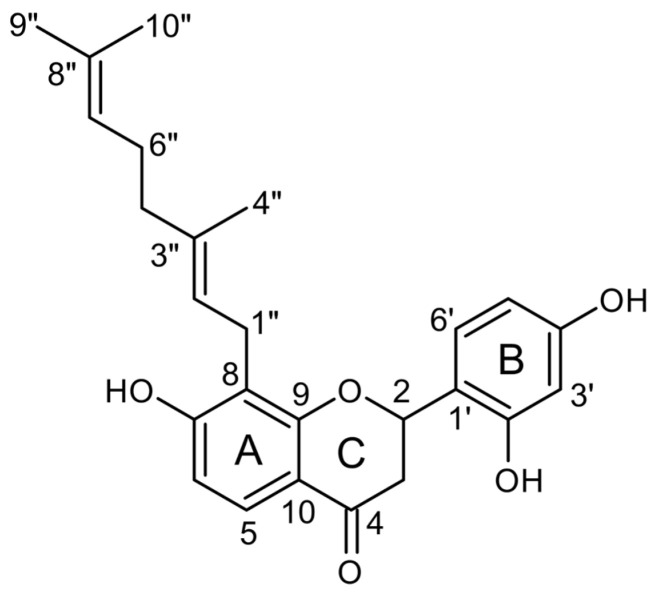
Chemical structure of malheuran A.

In this study, the successful establishment of a hairy root culture of *D. purpurea* is reported for the first time. A combination of different elicitors, including methyl-β-cyclodextrin (CD), hydrogen peroxide (H_2_O_2_), magnesium chloride (MgCl_2_), and methyl jasmonate (MeJA), was used for distinct periods to optimize the production of specialized metabolites, including the geranylated flavanone malheuran A (Figure 1). Additionally, malheuran A was isolated from the elicited hairy root culture of *D. purpurea* using normal and reversed-phase column chromatography and semi-preparative high-performance liquid chromatography (HPLC). The structure of malheuran A was determined based on nuclear magnetic resonance (NMR) and mass spectrometry (MS) data. This study describes the hairy root establishment of *D. purpurea* and the isolation, structure determination, and biological activities of malheuran A.

## 2. Results and Discussion

### 2.1. Establishment and Characterization of D. purpurea Hairy Root Cultures

**Figure 2:**
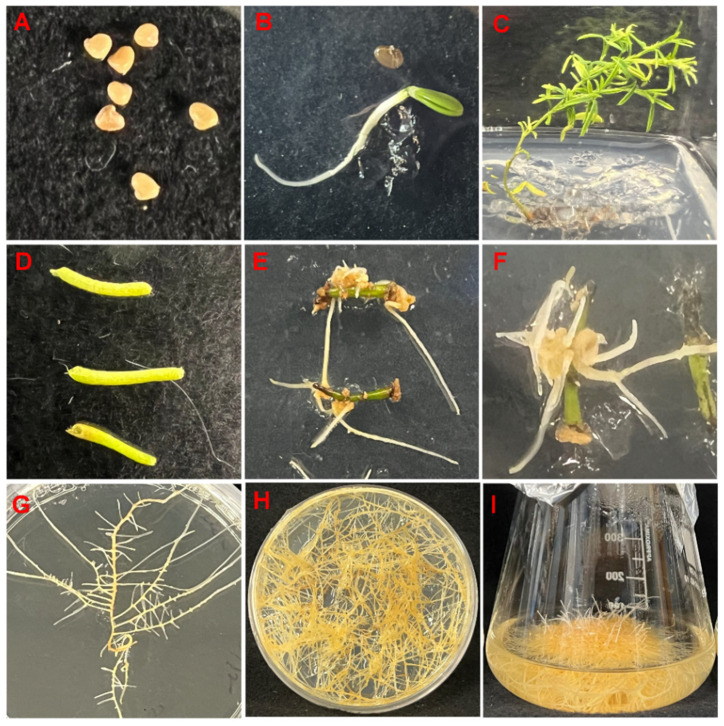
Establishment of hairy root cultures of *D. purpurea*. (**A**): Seeds of *D. purpurea*; (**B**): seven-day-old seedling; (**C**): sixty-seven-day-old seedling; (**D**): internodes (explants) from sixty-seven-day-old seedlings; (**E**,**F**): hairy root development from internodes infected with *A. rhizogenes* strain ATCC 15834; (**G**,**H**): growth of hairy roots on MSV media; (**I**): growth of hairy roots in the liquid medium after 15 days.

To induce hairy roots in *D. purpurea,* internodes from approximately 67-day-old in-vitro-grown seedlings were infected with *Agrobacterium rhizogenes* strain 15834, containing the root-inducing plasmid pRi15834. Hairy roots began to emerge around 43 days post-infection from the cut surfaces. About eight hairy roots were harvested, and the lines DP-1, DP-3, and DP-7 were chosen for further study based on their consistent growth. DP-1 was ultimately selected due to its superior growth characteristics in a liquid culture medium (Figure 2). It demonstrated continuous and robust growth compared to DP-3 and DP-7, making it the most promising candidate.

**Figure 3:**
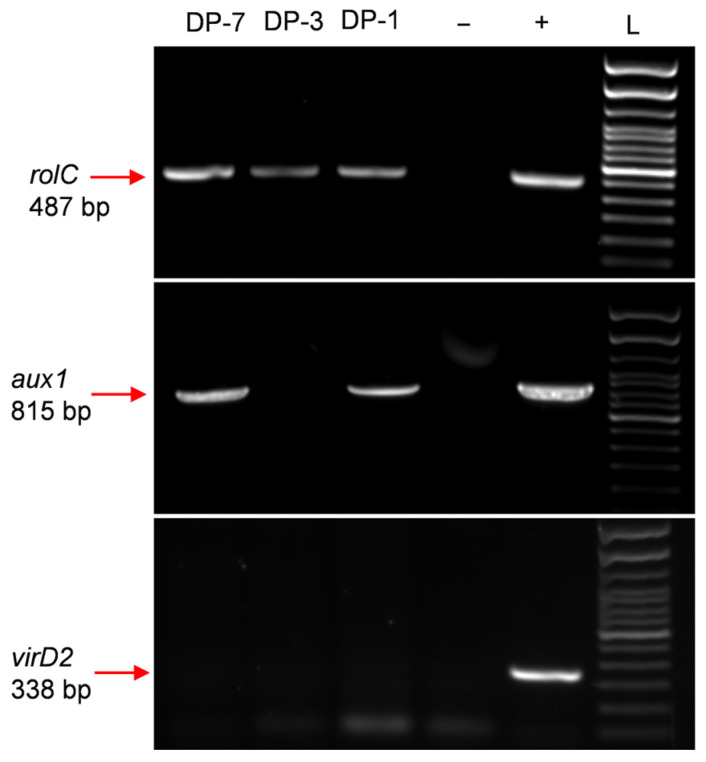
PCR analysis of selected *D. purpurea* hairy root lines. Hairy root lines DP-1, DP-3, and DP-7 were used for genomic DNA isolation. Primers targeting the *rolC*, *aux1*, and *virD2* genes were used for the analyses, with plasmid pRi15834 DNA as the positive control and double-distilled water (ddH_2_O) as the negative control. L: 100 bp DNA ladder.

PCR analysis on *D. purpurea* hairy root lines DP-1 and DP-7 confirmed the presence of the *rolC* and *aux1* genes, verifying the successful integration of the T-DNA from the Ri plasmid into the plant genome. PCR analysis for the *virD2* gene was negative, confirming that the hairy root tissue was free from *Agrobacterium*. In contrast, the DP-3 line exhibited the presence of the *rolC* gene but lacked both the *aux1* and *virD2* genes (Figure 3). The absence of the *aux1* gene in hairy root line DP-3 suggests compromised auxin biosynthesis, thereby impacting root development and broader physiology [29].

### 2.2. Growth Kinetics of D. purpurea Hairy Roots in 500 mL Flasks

**Figure 4:**
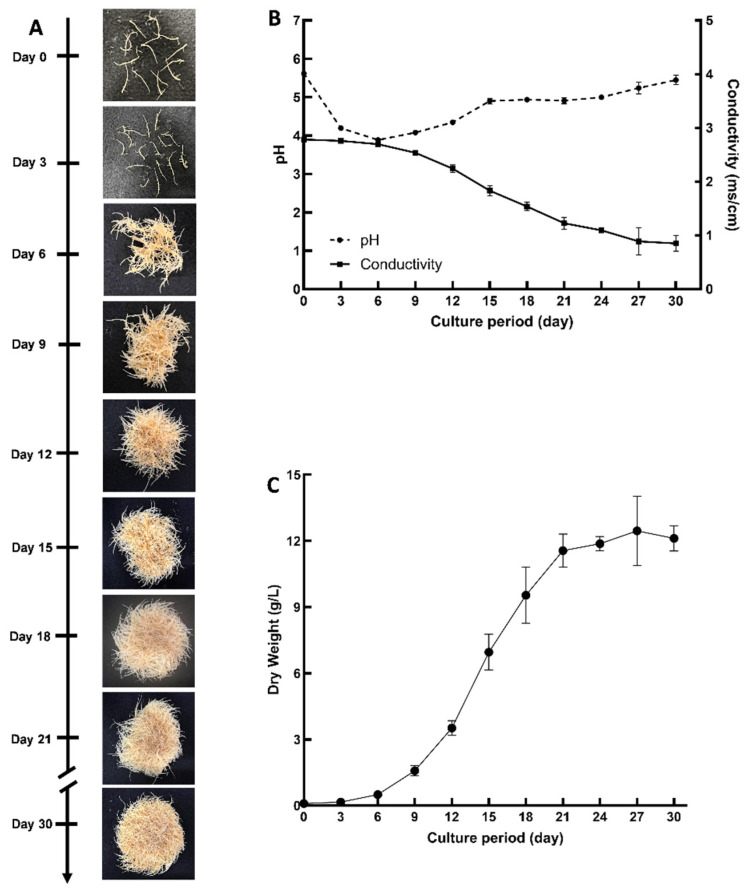
Growth of *D. purpurea* hairy root line DP-1. (**A**): Phenotype of *D. purpurea* hairy roots over the 30-day culture period, shown as representative images from three replicate cultures; (**B**): conductivity and pH of the medium at various growth stages; (**C**): growth curve of the hairy roots, with values representing the mean of three biological replicates; error bars indicate standard deviation.

Hairy roots of *D. purpurea* line DP-1 were grown in 500 mL flasks with 100 mL of MSV medium, and a thirty-day growth curve was constructed to determine the growth stages of the hairy roots grown under these conditions (Figure 4A). The pH and conductivity of the medium were measured over 30 days to analyze the behavior of the roots in the medium (Figure 4B). It was noted as the biomass increased, the conductivity of the medium decreased, suggesting that the hairy roots absorbed the nutrients from the culture medium during their growth. The pH decreased during the initial six days, gradually increased over the subsequent nine days, and then remained relatively constant until day 24. From day 27 until the end of the growth curve period, the pH exhibited a slow increase. Similar pH changes were observed in peanut hairy root cultures under comparable conditions [30]. Initially, the pH dropped during the first three days and then gradually increased over the next nine days, decreased again until day 18, and finally increased slowly until the end of the growth period. These results indicate that the pH of the medium initially decreases during the first few days and then slowly rises and gradually increases again towards the end of the growth period. A comparable pattern of conductivity and pH changes was also observed in peanut hairy root cultures [30]. Based on the dry weight of the hairy roots, a lag phase of approximately 3 days was observed, followed by an exponential growth phase from days 3 to 21 and a stationary phase from day 24 to 30 (Figure 4C). The specific growth rate, growth index, and doubling times were calculated as 0.23 day^−1^, 71.25, and 3 days, respectively. The maximum biomass achieved was 12.45 ± 1.57 g DW/L at 27 days. The specific growth rate was lower than that described for peanut hairy roots (0.31 day^−1^) grown in 500 mL flasks [30]. These findings suggest that *D. purpurea* hairy roots exhibit slower growth or biomass accumulation rates compared to peanut hairy roots under identical culture conditions.

### 2.3. Phenotype of Hairy Roots upon Elicitor Treatment

**Figure 5:**
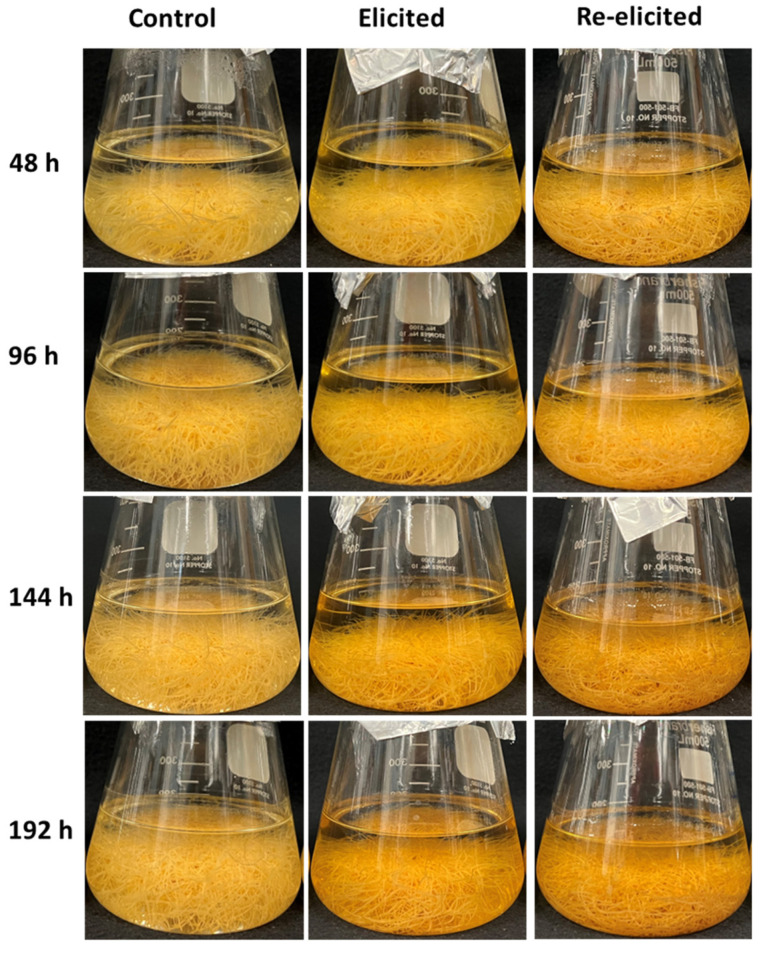
Changes in the phenotype of hairy root cultures of *D. purpurea* line DP-1 upon treatment with CD + H_2_O_2_ + MgCl_2_ + MeJA during a 192 h time course.

The study examined the impact of the combined elicitors CD + H_2_O_2_ + MgCl_2_ + MeJA on the production of malheuran A over a time ranging from 48 h to 192 h. *D. purpurea* hairy root cultures subjected to the treatment with CD + H_2_O_2_ + MgCl_2_ + MeJA exhibited the most pronounced color change following 192 h elicitation. The color of the culture medium changed from clear, as seen in the non-elicited control hairy root culture, to a pale-yellow color after elicitation, indicating the secretion of specialized metabolites into the medium (Figure 5). Interestingly, similar color changes were observed in previous studies involving peanut, pigeon pea, and mulberry hairy root cultures after treatment with a combination of CD + H_2_O_2_ + MgCl_2_ + MeJA [30,31,32]. These consistent observations underscore the significance of these treatments in the induction of specialized metabolites in various hairy root culture systems.

### 2.4. Effect of Elicitor Treatment on the Yield of Malheuran A in the Culture Medium

**Figure 6:**
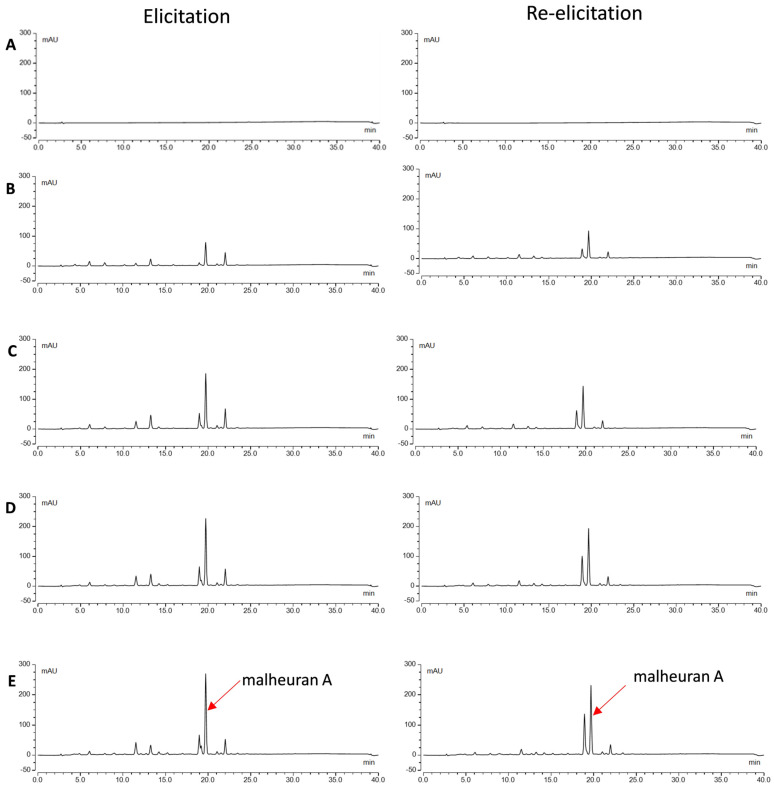
HPLC chromatograms of extracts from the medium of *D. purpurea* line DP-1 upon elicitation (**left**) and re-elicitation treatment with CD + H_2_O_2_ + MgCl_2_ + MeJA at (**A**): 192 h control (without elicitors); (**B**): 48 h; (**C**): 96 h; (**D**): 144 h; (**E**): 192 h. All chromatograms were monitored at 290 nm with the same scale −50 mAU to 300 mAU.

To assess the yield of malheuran A, *D. purpurea* hairy root cultures were treated with the combination of different elicitors CD + H_2_O_2_ + MgCl_2_ + MeJA for 192 h. Aliquots of the culture medium were collected at 48, 96, 144, and 192 h after elicitor treatment, extracted with ethyl acetate and analyzed by HPLC. Malheuran A was present in the elicited medium but not in the control medium (Figure 6). This suggests that malheuran A and other specialized metabolites in the culture media are inducible. They are secreted and accumulated in the medium in response to elicitor treatment, thus highlighting their potential as inducible metabolites within the culture system. Similar results were observed in peanut and pigeon pea hairy root cultures, where specialized metabolites were only detected in the culture medium after exposure to the stress induced by elicitors [30,31].

**Figure 7:**
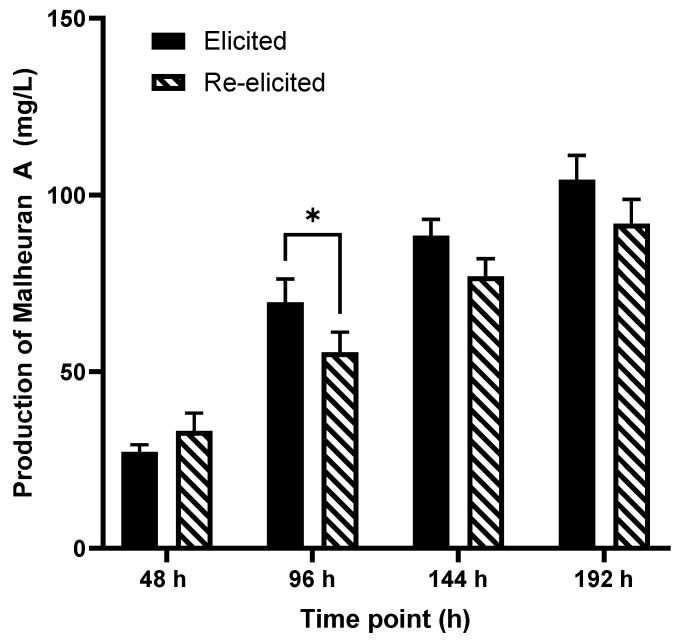
Comparison of malheuran A yield in elicited and re-elicited hairy root cultures of *D. purpurea* line DP-1 upon treatment with the elicitors CD + H_2_O_2_ + MgCl_2_ + MeJA. The yield is expressed as mg/L of medium. Each bar shows the average of three biological replicates, with error bars representing the standard deviation. Statistical analysis was performed using two-way ANOVA with Tukey’s multiple comparisons test (*, *p* < 0.05).

The lowest yield of malheuran A (27.2 ± 2.0 mg/L) was observed in the *D. purpurea* hairy root culture treated with CD + H_2_O_2_ + MgCl_2_ + MeJA for 48 h. The yield of malheuran A gradually increased with each time point: 96 h, 144 h, and 192 h. The highest amount of malheuran A (104.3 ± 6.9 mg/L) was observed 192 h after treatment, which was approximately 3.8-fold higher than the yield at 48 h (Figure 7). These results suggest that the production of malheuran A increases over time from 48 h to 192 h. Similarly, the production of prenylated stilbenoids increased over time from 48 h to 192 h in peanut hairy root cultures elicited with CD + H_2_O_2_ + MgCl_2_ + MeJA [30]. These findings suggest that 192 h is the optimum time to produce inducible metabolites in the hairy root culture systems of *D. purpurea* and peanut.

### 2.5. Yield of Malheuran A in the Hairy Root Culture System of D. purpurea

To investigate the overall total yield of malheuran A in the *D. purpurea* hairy root culture system, 18-day-old cultures were elicited for 192 h. Malheuran A was detected in the elicited culture medium, elicited hairy root tissue, and non-elicited hairy root tissue, but it was not present in the non-elicited control medium. The majority of malheuran A was produced and secreted in the elicited culture medium. The total yield of malheuran A present in the elicited medium and root tissues was determined per gram of the dry weight of the root.

**Figure 8:**
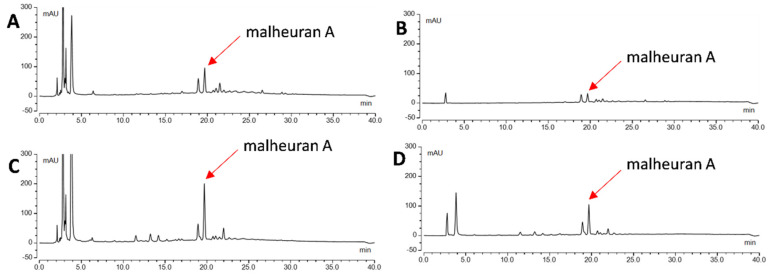
HPLC chromatograms of extracts from elicited hairy root tissues of *D*. *purpurea* line DP-1 extracted with 70% EtOH and EtOAC. (**A**): Control tissue extracted with 70% EtOH; (**B**): control tissue extracted with EtOAC; (**C**): elicited tissue extracted with 70% EtOH; (**D**): elicited tissue extracted with EtOAC. Elicitation was carried out for 192 h with CD + H_2_O_2_ + MgCl_2_ + MeJA. All chromatograms were monitored at 290 nm with the same scale −50 mAU to 300 mAU.

**Figure 9:**
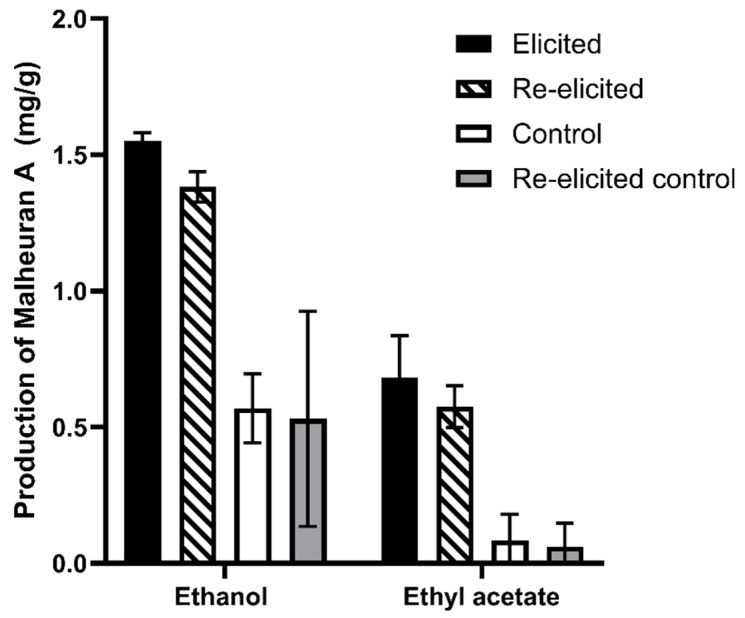
Comparison of malheuran A yield in hairy root tissues of *D. purpurea* line DP-1 extracted with 70% ethanol and ethyl acetate. The yield is expressed in mg/g DW hairy root tissue. Each bar represents the average of three biological replicates, and error bars represent the standard deviation.

To determine the yield of malheuran A in control and elicited hairy root tissues, the tissues were extracted with ethyl acetate and 70% ethanol. The objective was to determine the most efficient solvent for the extraction of malheuran A from the tissue. The results revealed that 70% ethanol was better than ethyl acetate for the extraction of malheuran A from hairy root tissues (Figure 8). The yield of malheuran A in elicited hairy root tissue extracted with 70% ethanol was 1.55 ± 0.02 mg/g DW, whereas the yield of malheuran A in elicited hairy root tissue extracted with ethyl acetate was 0.68 ± 0.15 mg/g DW (Figure 9).

**Figure 10:**
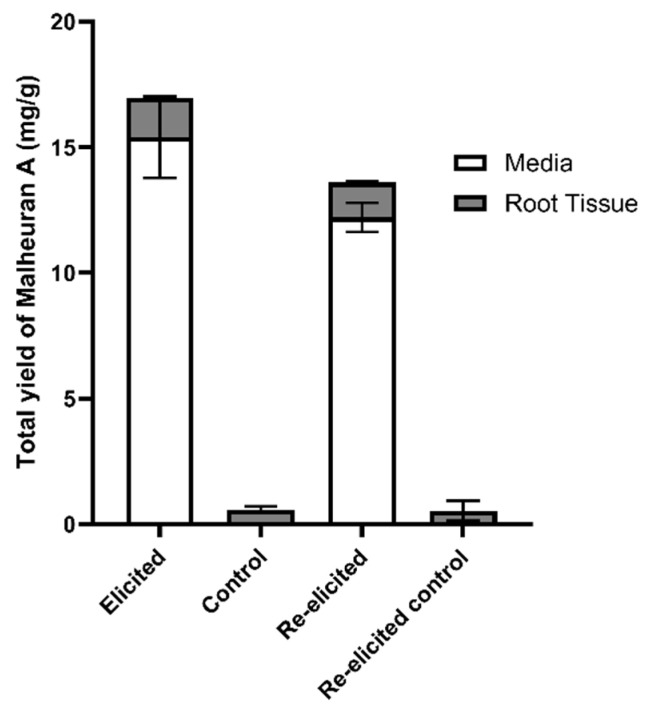
Total yield of malheuran A in the hairy root culture of *D. purpurea* line DP-1. Eighteen-day-old hairy root cultures were elicited with CD + H_2_O_2_ + MgCl_2_ + MeJA for 192 h, and then the elicited hairy roots were re-elicited for an additional 192 h. Each bar represents the average of three biological replicates, and error bars represent standard deviation. The yield is expressed in mg/g DW root.

The total yield of malheuran A in elicited culture media and root tissue of 18-day-old cultures elicited for 192 h was 16.95 ± 0.81 mg/g DW root. Intriguingly, the total yield of malheuran A in the elicited culture system was approximately 30-fold higher than in the non-elicited culture system. About 91% of malheuran A was secreted into the culture media of the elicited hairy root culture (Figure 10). This result suggests that after the stress induced by elicitors, most of the malheuran A was secreted into the culture medium. In a previous study, similar outcomes were observed when examining pigeon pea hairy root cultures. Extraction with 70% ethanol consistently yielded higher quantities of a prenylated flavonoid, isowighteone, compared to ethyl acetate extraction. Additionally, approximately 96% of isowighteone was secreted into the culture medium of elicited pigeon pea hairy root cultures [31].

### 2.6. Re-Elicitation of Elicited Hairy Root Cultures

The re-elicitation of *D. purpurea* hairy root cultures was conducted to assess the viability of the elicited hairy root cultures. After the initial 192 h elicitation period, the *D. purpurea* hairy root cultures were re-elicited for another 192 h using a combination of CD + H_2_O_2_ + MgCl_2_ + MeJA. The elicited hairy roots exhibited a robust response to the second round of elicitor treatment during re-elicitation and produced malheuran A and other specialized metabolites in the culture medium. The color of the re-elicited medium was changed to pale yellow (Figure 5). The highest yield of malheuran A in the culture medium (91.9 ± 6.8 mg/L) was observed at 192 h after re-elicitation, whereas the lowest yield (31.1 ± 5.0 mg/L) was observed at 48 h after re-elicitation (Figure 7). The yield of malheuran A in the elicited culture medium was approximately 1.1-fold higher compared to the yield in the re-elicited culture medium at 192 h. Similar results were obtained when peanut hairy root cultures were re-elicited with the same elicitors. The re-elicited hairy root cultures of peanut displayed a favorable response to elicitor treatment and produced a substantial amount of the prenylated stilbenoids arachidin-1 and arachidin-3 in the re-elicited culture medium [30].

**Figure 11:**
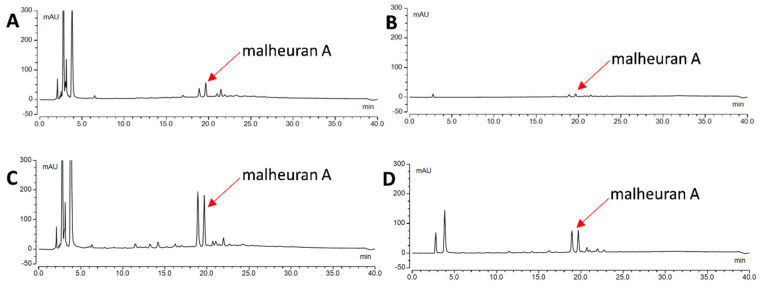
HPLC chromatograms of extracts from re-elicited hairy root tissues of *D. purpurea* line DP-1. (**A**): Control tissue extracted with 70% EtOH; (**B**): control tissue extracted with EtOAC; (**C**): re-elicited tissue extracted with 70% EtOH; (**D**): re-elicited tissue extracted with EtOAC. All chromatograms were monitored at 290 nm with the same scale −50 mAU to 300 mAU. Re-elicitation was carried out with CD + H_2_O_2_ + MgCl_2_ + MeJA for 192 h.

Both re-elicited and control hairy root culture tissues were extracted with 70% ethanol and ethyl acetate. Our results showed that 70% ethanol (1.38 ± 0.05 mg/g) was more effective for extraction compared to ethyl acetate (0.57 ± 0.07 mg/g) for re-elicited hairy root tissues (Figure 11). This outcome demonstrates the effectiveness of 70% ethanol in the extraction of malheuran A from elicited and re-elicited hairy root tissues. The total yield of malheuran A in the re-elicited culture media and root tissue of 192 h elicited cultures that were re-elicited for an additional 192 h was 13.58 ± 0.31 mg/g. Intriguingly, the total yield of malheuran A in the re-elicited culture system was approximately 26-fold higher than in the non-elicited culture system. About 90% of malheuran A was also secreted into the culture media of the re-elicited hairy root cultures (Figure 10).

The biomass of the hairy roots was higher in the re-elicited hairy root culture (1.5 ± 0.08 g DW) compared to the elicited culture (1.3 ± 0.05 g DW), representing an approximately 1.1-fold increase in the biomass of the re-elicited culture. Our results highlight the robust responsiveness of elicited *D. purpurea* hairy root cultures to re-elicitation. This observation underscores their suitability for repeated elicitation treatments, indicating a resilient metabolic activity and growth potential under such conditions. This re-elicitation approach can significantly reduce the time and production cost of specialized metabolites and help to obtain sufficient extract to streamline the purification process of individual metabolites, which can be utilized in various biomedical studies.

### 2.7. Purification of Malheuran A

**Figure 12:**
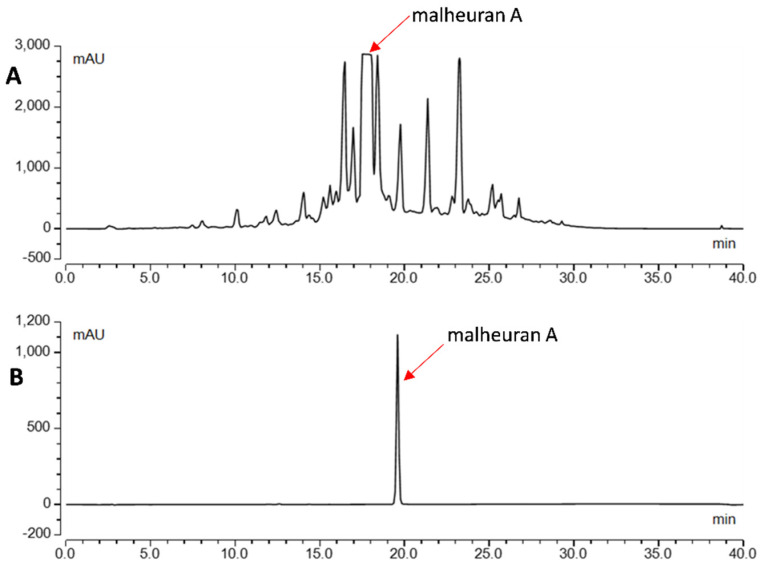
Semi-preparative HPLC purification and purity check of malheuran A. (**A**): Semi-preparative HPLC chromatogram of 7:3 (MeCN:0.5% HCOOH) octadecylsilane (ODS) fraction from hairy root cultures of *D. purpurea* elicited with CD + H_2_O_2_ + MgCl_2_ + MeJA; (**B**): analytical HPLC chromatogram of purified malheuran A (>95%) from hairy root cultures of *D. purpurea* line DP-1 elicited with CD + H_2_O_2_ + MgCl_2_ + MeJA. Detection was carried out at 290 nm.

Our study revealed that when the *D. purpurea* hairy root culture was elicited with CD + H_2_O_2_ + MgCl_2_ + MeJA for 192 h, it produced and secreted the highest amount of malheuran A into the culture medium. Consequently, the hairy root line DP-1 was cultured in MSV medium for eighteen days and then elicited with CD + H_2_O_2_ + MgCl_2_ + MeJA for 192 h. The elicited culture medium was then extracted with ethyl acetate, and the extract was sequentially fractionated using normal- and reversed-phase column chromatography. This was followed by semi-preparative reversed-phase HPLC purification, yielding 146 mg of purified malheuran A. To our knowledge, this is the first report detailing the isolation and purification of malheuran A in such a substantial quantity. A previous study also successfully isolated prenylated stilbenes arachidin-1 and arachidin-3 in large quantities from the hairy root culture of peanut [30]. These results indicate that elicited hairy root cultures can serve as a controlled production platform for inducible and secreted natural products on a large scale. By combining traditional column chromatography and semi-preparative HPLC, it becomes feasible to obtain bioactive natural products in large quantities when the extract is derived from elicited hairy root cultures. The purity of malheuran A was assessed using HPLC-UV based on the relative area covered by it. It was obtained at over 95% purity (Figure 12).

### 2.8. Identification of Malheuran A

Malheuran A was identified by comparing ^1^H and ^13^C NMR shifts, as well as MS data with values documented in the literature [33]. The UV absorption maxima at 207 and 287 nm were indicative of the presence of a flavanone skeleton [28]. Briefly, ^13^C NMR spectra revealed 25 carbons resonance, consisting of 15 characteristic carbon signals of a flavanone skeleton. This comprised one oxymethine (*δ*_C_ 75.0/*δ*_H_ 5.66), one methylene group with magnetically nonequivalent protons (*δ*_C_ 43.0/*δ*_H_ 2.68, 2.91), and one ketocarbonyl (*δ*_C_ 190.7) (Table 1). The ^1^H and ^13^C NMR data indicated the presence of geranyl chain, which included three methyl singlets (δ_H_ 1.50, 1.56, and 1.63), three methylenes (δ_H_ 1.90, 2.02, and 3.33), and two methine groups (δ_H_ 5.02 and 5.25), and two quaternary olefinic carbon signals (*δ*_C_ 130.7 and 134.5). The observed HMBC correlations from H_2_-1″ to C-7, C-8, and C-9 and from H-2″ to C-8 indicated the location of the geranyl group at C-8 of ring A. The overall structure was established by the comparison of 1D and 2D NMR spectroscopic data to values reported in the literature [33] (Figures S1–S6, Supporting Information).

Malheuran A was further corroborated by MS data taken in both positive and negative ion modes. The molecular ions observed in positive ESI-MS at *m*/*z* 409.33 [M + H]^+^ and in negative ESI-MS at *m*/*z* 407.63 [M − H]^−^ enabled the deduction of its molecular weight at 408 Da (Table 2). The fragment ion at *m*/*z* 273 in MS^2^ in positive ion mode was indicative of the loss of the geranyl side chain from the parent flavonoid structure (Figures S7 and S8, Supporting Information).

Malheuran A has been isolated previously from *D. searlsiae* and *D. ornata* [12,33], with this study marking its first isolation from *D. purpurea*. While its antimicrobial, insecticidal, and anthelmintic activities have been studied [12,33], our investigation introduces the novel exploration of its anti-inflammatory potential, alongside expanded testing against additional bacterial strains. To date, three geranylated flavonoids, including malheuran A, have been identified in *Dalea* species. Furthermore, the isolation of malheuran D and prostratol F among other geranylated flavonoids from *Dalea* species suggests an underexplored potential in this group [33]. These findings highlight the yet untapped richness of geranylated flavonoids in *Dalea* species, presenting promising avenues for compound discovery through *Dalea* hairy root cultures.

### 2.9. Antimicrobial Activity of Malheuran A

All biological assays were performed with the purified malheuran A. The antimicrobial activity of malheuran A was evaluated against Gram-positive and Gram-negative bacteria using a microculture method. The compound exhibited strong antimicrobial activities against Gram-positive bacteria with MIC values ranging from 1.56 to 6.25 μg/mL (Table 3). However, it did not show appreciable activity against Gram-negative bacteria with MIC values > 100 μg/mL. Malheuran A displayed potent activity against both methicillin-resistant *Staphylococcus aureus* (MRSA) and vancomycin-resistant Enterococci (VRE) with MIC values of 3.12 µg/mL. Consistent with prior research, a previous study revealed that malheuran A effectively inhibited the growth of both oxacillin-sensitive and resistant *S. aureus* with MIC values ranging from 3.7 to 4.2 μg/mL. Our study expanded upon these findings by including bacterial strains not previously tested against malheuran A such as *Enterococcus faecalis* ATCC 29212, *E. faecium* ATCC 700221 (VRE), *E. faecalis* ATCC 51299 (VRE), *Bacillus subtilis* ATCC 6623, *Micrococcus luteus* ATCC 10240, and *S. epidermidis* ATCC 700296. Malheuran A demonstrated potent activity against *E. faecalis* ATCC 29212 and VRE strains with MIC values of 3.12 µg/mL. Additionally, malheuran A exhibited strong activity against *M. luteus* ATCC 10240, *B. subtilis* ATCC 6623, and *S. epidermidis* ATCC 700296 with MIC values ranging from 1.56 to 6.25 µg/mL. The antimicrobial activity of malheuran A may be attributed to the presence of a geranyl moiety. The geranyl moiety potentially enhances the lipophilicity of the molecule, thereby increasing its antimicrobial efficacy through improved penetration into microbial cells [34]. Additionally, the presence of three phenolic hydroxyl groups may also enhance the antimicrobial activity of malheuran A. In a previous study, flavonoids with prenyl chains containing two or three phenolic hydroxyl groups showed potent antimicrobial activity against VRE, relative to those with other structural features [35]. *S. aureus* and Enterococci are the leading causes of nosocomial infections in healthcare settings. Moreover, according to various reports, infections caused by MRSA and VRE have been increasing in hospitals [24,25]. Therefore, there is an urgent need to find natural products that can control infections caused by such types of multi-drug-resistant microorganisms. Natural products could serve as promising candidates for the prevention and treatment of MRSA and VRE infections. Our findings highlight the significant activity of malheuran A against MRSA and VRE, suggesting its potential as a promising candidate for treating infectious bacteria such as MRSA and VRE in the medical field.

The production of plant specialized metabolites such as malheuran A in *D. purpurea* may serve as a vital defense mechanism against microbial pathogens and environmental stress. Our study reveals that malheuran A production can be induced by exposing the hairy root cultures to stress caused by a combination of chemical elicitors. In nature, stress can include microbial pathogens, ultraviolet radiation, and chemical compounds. This emphasizes the adaptive strategy of plants to combat diverse environmental challenges.

In their natural habitat, plants encounter a range of stresses, and the production of specialized metabolites such as malheruan A in response to these challenges might suggest an ability to adapt and survive in hostile environments [36].

### 2.10. Anti-Inflammatory Activity of Malheuran A

**Figure 13:**
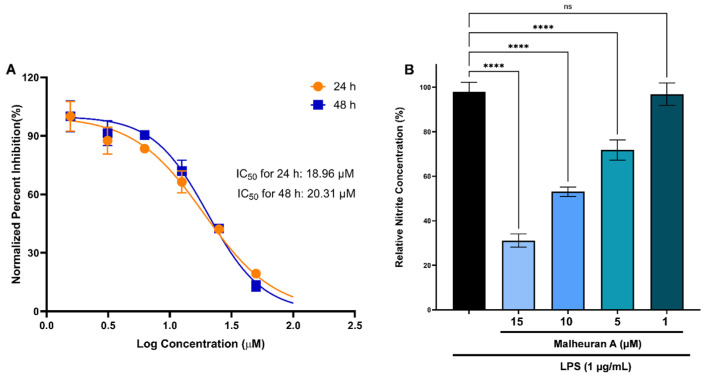
Cytotoxicity and anti-inflammatory activity of malheuran A. (**A**): Cytotoxicity of malheuran A on RAW 264.7 macrophages; (**B**): nitrite production by LPS-stimulated RAW 264.7 cells pre-treated with or without malheuran A at different concentrations. Each bar represents the average of three biological replicates, and error bars represent standard deviation. Statistical analysis was performed using Brown–Forsythe one-way analysis of variance (ANOVA) with Dunnett’s post-test. The asterisks above the connecting lines represent a significant difference when compared to nitrite level in LPS-only-treated RAW 264.7 cells (****, *p* < 0.0001; ns, not significant).

The cytotoxic effects of malheuran A on RAW 264.7 macrophages were evaluated in vitro using the RealTime-Glo™ MT Cell Viability Assay over 48 h at doses ranging from 1.56 to 100 µM. The IC_50_ values of malheuran A in RAW 264.7 cells were found to be 18.96 µM at 24 h and 20.31 µM at 48 h (Figure 13A).

Nitrite levels were determined as an indicator of nitric oxide production. Thus, to assess the impact of malheuran A on nitrite levels after LPS treatment, the Griess Reagent System was utilized. During the incubation period, malheuran A notably decreased nitrite levels at concentrations of 15, 10, and 5 µM compared to cells treated only with LPS. Specifically, the nitrite levels observed in cells treated with 15, 10, and 5 µM malheuran A followed by LPS treatment were 31.11 ± 2.97%, 53.08 ± 2.11%, and 71.83 ± 4.53%, respectively, relative to cells treated solely with LPS (Figure 13B). These findings suggest the potent anti-inflammatory activity of malheuran A at low micromolar concentrations.

## 3. Materials and Methods

### 3.1. Seed Sterilization and Germination of D. purpurea

Seeds *of D. purpurea* were sourced from the Genetic Resources Information Network (GRIN)-Global (accession number: PI 599339). Surface sterilization of the seeds was performed using 0.1% Palmolive^®^ detergent for 2 min followed by 50% Clorox^®^ for 15 min. Subsequently, the seeds were washed 5–6 times with sterile distilled water and cultured on MS [37] medium supplemented with 3% sucrose (pH 5.7) and 0.4% phytagel. The seeds were incubated in the dark until germination. Seeds were considered germinated when radicle emergence was visible. Germinated seeds were placed in Phytatray™ boxes (Millipore Sigma, St. Louis, MO, USA) containing MS medium and incubated at 24 °C in a photoperiod incubator with a 16 h light/8 h dark cycle.

### 3.2. Establishment of Hairy Root Lines of D. purpurea

The internodes served as explants for hairy root induction. Internodes were harvested from 67-day-old seedlings of *D. purpurea* and wounded with a scalpel containing *Agrobacterium rhizogenes* strain ATCC 15834. The internodes were subsequently cultured on modified Murashige and Skoog’s medium (referred to as MSV medium [38]). The cultured plates were incubated for 3 days until *Agrobacterium* growth became evident on the internodes. The internodes were then placed on MSV medium supplemented with 250 mg/L of cefotaxime to kill the *Agrobacterium*. In approximately 43 days, hairy roots developed from the cut surfaces. Then, the hairy roots were removed from the internodes, transferred on MSV medium with 150 mg/L of cefotaxime for 2–3 weeks, and then placed on MSV medium without any antibiotics. Among several lines of *D. purpurea* hairy roots, line DP-1 was selected for further study due to its robust and sustained growth. The successful establishment of hairy root cultures was validated by performing PCR analysis for the *rolC*, *aux1*, and *virD2* genes, as described in earlier work [39]. The DNeasy Plant Mini Kit (Qiagen, Germantown, MD, USA) was used to isolate genomic DNA.

### 3.3. Growth Kinetics of D. purpurea Hairy Roots

To establish a growth curve, fifteen tips 2–3 cm in length were cut from the hairy roots of line DP-1 cultured in MSV medium and transferred to 500 mL flasks containing 100 mL of MSV liquid medium supplemented with 3% sucrose. The flask cultures were placed on an orbital shaker (Innova 44R, New Brunswick Scientific, Hauppauge, NY, USA) at 90 rpm and 28 °C in continuous darkness. Every three days, three flasks were collected until day 30. After harvesting, the hairy roots were rinsed with tap water and then frozen and lyophilized (Freeze Dry System Freezone 4.5, Labconco™, Kansas City, MO, USA) to determine the dry weight. The specific growth rate (µ) was calculated using the formula µ = ln (DW_i_/DW_0_)/Δt where DW_i_ = 11.56 g/L represents the average dry weight of the roots at the end of the exponential phase (day 21), DW_0_ = 0.16 g/L is the average dry weight at the start of the exponential phase (day 3), and t is the interval of time (in days) between 3 and 21 (18 days). Doubling time (T_d_) was calculated as T_d_ = ln (2)/µ. The growth index (GI) was determined as GI = (DW_i_ − DW_0_)/DW_0_ where DW_i_ is the average dry weight of the roots at day 21, and DW_0_ is the average dry weight of inoculum at day 0. The conductivity and pH measurements were taken from the culture media at each time point.

### 3.4. Elicitation of D. purpurea Hairy Root Culture and Analytical HPLC Analysis

Hairy roots of *D. purpurea* line DP-1 were cultured in 500 mL flasks containing 100 mL of MSV medium. On day 18, the MSV medium was discarded, and the hairy roots were elicited with fresh MSV medium supplemented with 3% sucrose containing a combination of elicitors: 125 μM methyl jasmonate (MeJA; Sigma-Aldrich, St. Louis, MO, USA), 18 g/L methyl-β-cyclodextrin (CD; CAVASOL^®^ W7 M, Wacker, Munich, Germany), 3 mM hydrogen peroxide (H_2_O_2_; Thermo Scientific, Waltham, MA, USA), and 1 mM magnesium chloride (MgCl_2_; Sigma-Aldrich, St. Louis, MO, USA). All elicited cultures were incubated at 28 °C in darkness on a rotary shaker set to 90 rpm. Fresh MSV medium without elicitors was added to the control group. At 48 h intervals, aliquots of the elicited medium were collected from the same flasks between 48 and 192 h to assess the metabolic profile at different time points using HPLC. The elicitation was conducted in biological triplicates.

Analytical HPLC analysis was conducted in an Ultimate 3000 ultra-high-performance liquid chromatography (UHPLC) system (Thermo Fisher Scientific, Waltham, MA, USA). The chromatography was carried out on a SunFire^TM^ C18 column (5 µm, 4.6 × 250 mm, UV detection at 290 nm) (Waters, Milford, MA, USA) at 40 °C and a 1.0 mL/min flow rate. The mobile phase was composed of 0.5% HCOOH (A) and MeOH (B) (*v*/*v*). Separation was achieved by HPLC with a gradient of MeOH from 50% B to 100% B for 0–30 min, 100% B for 30–35 min, and 50% B for 35–40 min. Similarly, re-elicited culture media were analyzed by HPLC. Dilutions of purified malheuran A were prepared in MeOH to generate calibration curves for quantitative analysis. Calibration curves were constructed using absorbance at 290 nm (y = 0.3679x + 1.2716, LOQ = 37.20 mg/L, LOD = 12.27 mg/L, R^2^ = 0.9995).

### 3.5. Re-Elicitation of D. purpurea Hairy Root Cultures

To assess the capacity of the elicited hairy roots to produce metabolites upon elicitor treatment, the elicited hairy root cultures were re-elicited with the same combination of elicitors used during the first elicitation. All the re-elicited cultures were placed on a rotary shaker at 90 rpm and incubated at 28 °C under continuous darkness. Aliquots of the medium were collected from the same flask of the re-elicited culture at various time points 48, 96, 144, and 192 h for the time-course experiment and analyzed by HPLC analysis. A control group was examined by supplementing fresh MSV medium without the addition of elicitors. The re-elicitation was conducted in biological triplicates.

### 3.6. Extraction and HPLC Analysis of Polyphenols from D. purpurea Hairy Root Culture Medium and Tissues

To analyze the yield of malheuran A at each time point from 48 to 192 h, a 900 µL aliquot of the elicited medium was collected. Polyphenols were extracted by adding an equal volume of ethyl acetate, followed by vortexing for 30 s. The mixture was then centrifuged for 10 min at 10,000 rpm. The upper organic layer was transferred to an HPLC vial and dried in vacuo. The dried extract was resuspended in 500 µL of methanol and analyzed using reversed-phase HPLC. The re-elicited culture medium was processed and analyzed in the same manner.

Hairy root tissues after 192 h of elicitation were frozen and then lyophilized to extract the phenolic compounds as follows. The dried lyophilized root tissue was ground into a fine powder with a mortar and pestle. Two solvents, 70% ethanol and ethyl acetate, were used to extract compounds from the root tissues. For extraction, 0.1 g of root tissue was mixed with 2 mL of 70% ethanol or ethyl acetate and sonicated for 10 min. Subsequently, it was centrifuged at 3220× *g* for 10 min, and the supernatant was collected and analyzed by HPLC. Similarly, re-elicited and control root tissues were extracted and analyzed by HPLC.

### 3.7. Extraction and Isolation of Malheuran A

*D. purpurea* hairy root line DP-1 maintained on MSV medium was used for large-scale elicitation to obtain an adequate amount of extract for malheuran A isolation. Hairy roots were initially inoculated into twenty 500 mL flasks containing 100 mL of MSV medium. The cultures were allowed to grow until reaching the mid-log phase, at which point the medium was replaced with 200 mL of fresh MSV medium containing 3% sucrose, 18 g/L CD, 125 μM MeJA, 3 mM H_2_O_2_, and 1 mM MgCl_2_. The cultures were then incubated at 28 °C on a rotary shaker at 90 rpm in continuous darkness for 192 h. At the end of 192 h elicitation, the elicited culture media were harvested and extracted with ethyl acetate. The organic layer was separated using a separatory funnel, and the combined organic layers were concentrated in vacuo, yielding 2.5 g of extract from 4 L of elicited culture medium.

Malheruan A was purified according to a previously established method [30]. The extract was separated using silica gel column chromatography (Sigma-Aldrich, St. Louis, MO, USA) with a stepwise gradient of CHCl_3_–MeOH (1:0, 20:1, 10:1, 4:1, 2:1, 1:1, and 0:1 *v*/*v*). Fraction 4 (4:1) was concentrated, yielding 1.15 g of yellowish oil. This fraction was further fractionated using reversed-phase octadecylsilane (ODS) column chromatography (Nacalai Tesque, Inc., Kyoto, Japan) with a gradient of MeCN-0.5% HCOOH (2:8, 3:7, 4:6, 5:5, 6:4, 7:3, and 8:2 *v*/*v*). Fraction 6 (7:3) was then concentrated and extracted with ethyl acetate. The organic phase was dried over anhydrous Na_2_SO_4_, filtered, and concentrated to obtain 328 mg of semi-purified material. Final purification was achieved by semi-preparative HPLC (SunFire C18 OBD™ Prep, Waters, Milford, MA, USA) (10 × 250 mm, 4 mL/min, UV detection at 290 nm) with gradient elution of MeCN/0.5% HCOOH solution (35:65 to 70:30 for 20 min and then 70:30 to 100:0 over 30 min) to yield malheuran A (146 mg, *t*_R_ 17.5 min). Malheuran A was obtained with >95% purity, as determined by HPLC-UV based on the relative area covered by the compound peak.

### 3.8. Nuclear Magnetic Resonance Spectrometer (NMR) Analysis

NMR spectra were obtained on a JEOL spectrometer at 400 MHz for ^1^H and 101 MHz for ^13^C in acetone-*d*_6_. The signals for residual solvent (*δ*_H_ 2.02) and (*δ*_C_ 29.0, 205.5) were used as internal standards.

### 3.9. Liquid Chromatography–Mass Spectrometry (LC–MS) Analysis

HPLC separation was carried out using the UltiMate 3000 UHPLC system (Thermo Scientific, Waltham, MA, USA). The separation method adhered to the HPLC conditions described above, except for using 0.1% formic acid. Mass spectrometry was conducted using an LTQ XL linear ion trap mass spectrometer (Thermo Scientific, Waltham, MA, USA) equipped with an electrospray ionization (ESI) source. MS parameters were used as previously described [40]. Mass spectra were acquired in both positive and negative ion modes, and full mass scans covered the range of *m*/*z* 110–1000. Data analysis was conducted using Xcalibur software version 4.4.16.14 (Thermo Scientific, Waltham, MA, USA).

### 3.10. Antimicrobial Assay

The antimicrobial activity of malheuran A was evaluated according to a protocol described previously [41]. Mueller–Hinton Broth (Difco) served as the medium for the antimicrobial assay, except for *Streptococcus mutans*, for which Brain Heart Infusion Broth (Difco) was used. Vancomycin hydrochloride for Gram-positive bacteria, daptomycin for vancomycin-resistant Enterococci (VRE), and colistin sulfate for Gram-negative bacterial strains were used as reference antibiotics. The test strains, including *Staphylococcus aureus* ATCC 25923, *Staphylococcus aureus* ATCC 35591 (MRSA), *Staphylococcus aureus* ATCC 00699 (MRSA), *Staphylococcus epidermidis* ATCC 700296, *Enterococcus faecalis* ATCC 29212, *Enterococcus faecalis* ATCC 51299 (VRE), *Enterococcus faecium* ATCC 700221 (VRE)*, Bacillus subtilis* ATCC 6623, *Escherichia coli* ATCC 25922, *Klebsiella pneumoniae* ATCC 700603, and *Acinetobacter baumannii* ATCC 747, were used to investigate antimicrobial activity of malheuran A. The plates were incubated at 37 °C for 48 h under aerobic conditions, except for *S. mutans,* which was incubated in a 5% CO_2_ incubator. The minimum inhibitory concentration (MIC) was defined as the lowest concentration at which microbial growth was completely inhibited.

### 3.11. Anti-Inflammatory Assay

#### 3.11.1. Cell Culture

The RAW 264.7 mouse macrophage cell line was obtained from the American Type Culture Collection (ATCC^®^ TIB-71™, ATCC, Manassas, VA, USA). The cells were cultured in Dulbecco’s Modified Eagle Medium (DMEM) supplemented with 10% fetal bovine serum (FBS, Cytiva-HyClone, Logan, UT, USA), 100 μg/mL streptomycin, and 100 U/mL penicillin in a humidified incubator (5% CO_2_) at 37 °C.

#### 3.11.2. Cytotoxicity Assay and Quantification of Nitrite Level

The effect of malheuran A on RAW 264.7 macrophage cell viability was assessed using the RealTime-Glo™ MT Cell Viability Assay (Promega, Madison, WI, USA), following the manufacturer’s instructions. Initially, 2000 cells/well were seeded in clear-bottom 96-well plates and allowed to culture overnight. Subsequently, the medium was replaced with varying concentrations of malheuran A from 1.56 to 100 µM along with the RealTime-Glo™ MT Cell Viability Assay reagents, which include the NanoLuc^®^ Luciferase and the MT Cell Viability Substrate. Luminescence was then measured at different time points using a BioTek Cytation 5 reader.

For nitrite level induction, RAW 264.7 cells were cultured in 24-well plates at a seeding density of 10^5^ cells/well overnight. The cells were pre-treated with malheuran A at concentrations of 15, 10, 5, and 1 µM for 2 h, followed by co-stimulation with LPS at 1 µg/mL for 24 h. Subsequently, the culture medium was collected, and nitrite levels were quantified using the Griess Reagent System (Promega, Madison, WI, USA), following the manufacturer’s protocol.

### 3.12. Statistical Analysis

Two-way ANOVA with Tukey’s multiple-comparison tests was performed for data in Figure 7, and Brown–Forsythe one-way analysis of variance (ANOVA) with Dunnett’s post-test was used for data in Figure 13 using GraphPad Prism 9 software (San Diego, CA, USA).

## 4. Conclusions

The scarcity of geranylated flavonoids in nature poses a significant challenge to their pharmaceutical applications. While chemical synthesis is complex and inefficient, alternative approaches, such as the utilization of hairy root cultures, offer a promising solution to overcome the supply issue. By employing a hairy root culture platform with elicitation and re-elicitation techniques, the production of geranylated flavonoids, such as malheuran A, can be substantially enhanced. Our HPLC-UV investigation of *D. purpurea* hairy root cultures revealed intriguing findings, including compounds resembling malheuran A and others with unique UV profiles, suggesting the presence of previously unidentified metabolites. These findings highlight the potential of the *D. purpurea* hairy root culture system for further research and exploration, offering a valuable avenue for the isolation and identification of new bioactive compounds. To fully harness this potential, scalable and cost-effective bioprocesses must be developed for cultivating hairy root cultures. Bioreactors or other innovative technologies can enable the production of metabolites on a commercial scale, facilitating their application in drug development and other industries.

## Figures and Tables

**Table 1:** ^1^H and ^13^C NMR data for malheuran A in acetone-*d*_6_.

Position	δ_C_ ^a^	δ_H_, Mult (*J* in Hz) ^b^	HMBC ^b,c^
2	75.0, CH	5.66, dd (13.0, 2.7)	4, 1′, 2′, 6′
3	43.0, CH_2_	2.91, dd (16.7, 13.0)	2, 1′, 4
		2.68, dd (16.7, 2.7)	4, 10
4	190.7, C		
5	125.5, CH	7.57, d (8.6)	4, 7, 9
6	109.5, CH	6.60, d (8.6)	8, 10
7	161.6, C		
8	115.5, C		
9	161.3, C		
10	114.5, C		
1′	117.5, C		
2′	155.2, C		
3′	102.5, CH	6.44, d (2.0)	1′, 5′, 2′, 4′
4′	158.4, C		
5′	106.9, CH	6.42, dd (8.3, 2.0)	3′, 1′, 4′
6′	127.8, CH	7.35, d (8.3)	2, 2′, 4′
1″	21.9, CH_2_	3.33, m	7, 8, 9, 2″, 3″
2″	122.3, CH	5.25, t (7.1)	5″, 1″, 4″, 8
3″	134.5, C		
4″	15.4, CH_3_	1.63, s	5″, 2″, 3″
5″	39.6, CH_2_	1.90, m	4″, 6″, 2″, 7″, 3″
6″	26.5, CH_2_	2.02 ^d^	5″, 8″, 7″, 3″
7″	124.3, CH	5.02, t (6.8)	6″, 10″, 9”
8″	130.7, C		
9″	25.0, CH_3_	1.56, s	8″, 7″, 10″
10″	16.9, CH_3_	1.50, s	8″, 7″, 9″

^a^ Recorded at 101 MHz (reference δ_C_ 29.0, 205.5). ^b^ Recorded at 400 MHz (reference δ_H_ 2.02). ^c^ HMBC correlations are from proton(s) stated to the indicated carbon. ^d^ Overlapped with the solvent signal.

**Table 2:** Mass spectrometry analysis of malheuran A detected in ethyl acetate extract from the medium of elicited *D. purpurea* line DP-1 hairy root culture. Analysis was conducted by HPLC-PDA-ESI-MS^2^.

*t*_R_ (min)	UV Max (nm)	[M + H]^+^	MS^2^ Ions	[M − H]^−^	MS^2^ Ions
19.4	207, 221 (sh), 233 (sh), 287, 322 (sh)	409	273, 391	407	160, 245, 389

*t*_R_: Retention time in minutes.

**Table 3:** Antimicrobial activity of malheuran A.

Microorganisms	MIC (µg/mL)	Reference (µg/mL)
*Acinetobacter baumannii* ATCC 747 ^a^	>100	0.62 ^c^
*Bacillus subtilis* ATCC 6623 ^b^	3.12	0.31 ^d^
*Enterococcus faecalis* ATCC 29212 ^b^	3.12	5 ^d^
*Enterococcus faecalis* ATCC 51299 (VRE) ^b^	3.12	10 ^e^
*Enterococcus faecium* ATCC 700221 (VRE) ^b^	3.12	5 ^e^
*Escherichia coli* ATCC 25922 ^a^	>100	0.31 ^c^
*Klebsiella pneumoniae* ATCC 700603 ^a^	>100	0.62 ^c^
*Micrococcus luteus* ATCC 10240 ^b^	1.56	0.31 ^d^
*Staphylococcus aureus* ATCC 25923 ^b^	3.12	1.25 ^d^
*Staphylococcus aureus* ATCC 35591 (MRSA) ^b^	3.12	2.5 ^d^
*Staphylococcus aureus* ATCC 00699 (MRSA) ^b^	3.12	5 ^d^
*Staphylococcus epidermidis* ATCC 700296 ^b^	6.25	2.5 ^d^
*Streptococcus mutans* UA159 ^b^	3.12	1.25 ^d^

^a^ Gram-negative bacteria. ^b^ Gram-positive bacteria. ^c^ Colistin sulfate. ^d^ Vancomycin hydrochloride. ^e^ Daptomycin.

## Data Availability

The data of this work are available upon request.

## Supplementary Materials

The following supporting information can be downloaded at https://www.mdpi.com/article/10.3390/plants14020259/s1. Figure S1. ^1^H NMR spectrum of malheuran A (400 MHz, acetone-*d*_6_); Figure S2. ^13^C NMR spectrum of malheuran A (101 MHz, acetone-*d*_6_).; Figure S3. DEPT135 spectrum of malheuran A (101 MHz, acetone-*d*_6_); Figure S4. COSY spectrum of malheuran A (400 MHz, acetone-*d*_6_); Figure S5. HSQC spectrum of malheuran A (400 MHz, acetone-*d*_6_); Figure S6. HMBC spectrum of malheuran A (400 MHz, acetone-*d*_6_); Figure S7. LC–MS analysis of malheuran A from CD + H_2_O_2_ + MgCl_2_ + MeJA elicited extract in positive ion mode. (A) HPLC chromatogram; (B) MS ion chromatogram; (C) MS^2^ ion chromatogram; Figure S8. LC–MS analysis of malheuran A from CD + H_2_O_2_ + MgCl_2_ + MeJA elicited extract in negative ion mode. (A) HPLC chromatogram; (B) MS ion chromatogram; (C) MS^2^ ion chromatogram.
