# Supplementary material for: Production of Malheuran A, a Geranylated Flavonoid with Antimicrobial and Anti-Inflammatory Activities, in Hairy Root Cultures of Dalea purpurea
Source: Plants (Basel). 2025 Jan 17;14(2):259. doi: 10.3390/plants14020259 (PMC11769231; doi:10.3390/plants14020259)
Supplement: Supplementary file 1 [file plants-14-00259-s001.zip › plants-3407573-supplementary.pdf]

Supporting information

Production of malheuran A, a geranylated flavonoid with antimicrobial and anti-inflammatory activities, in hairy root cultures of *Dalea purpurea*

Amit Raj Sharma[1], Gaurav Gajurel[1,2], Salma Abdel-Karim[1], Mohammad A. Alam[3], Robert Colquhoun Shields[4], and Fabricio Medina-Bolivar[1,4*]

[1]Arkansas Biosciences Institute, Arkansas State University, Jonesboro, AR 72401, USA

[2]Molecular Biosciences Graduate Program, Arkansas State University, Jonesboro, AR 72401, USA

[3]Department of Chemistry and Physics, Arkansas State University, Jonesboro, AR 72401, USA

[4]Department of Biological Sciences, Arkansas State University, Jonesboro, AR 72401, USA

* Corresponding author:

Fabricio Medina-Bolivar, Ph,D.

Arkansas Biosciences Institute, Arkansas State University, P.O. Box 639, State University, AR 72467, USA; Phone: +18706804319; Fax: +18706804348; E-mail: fmedinabolivar@astate.edu

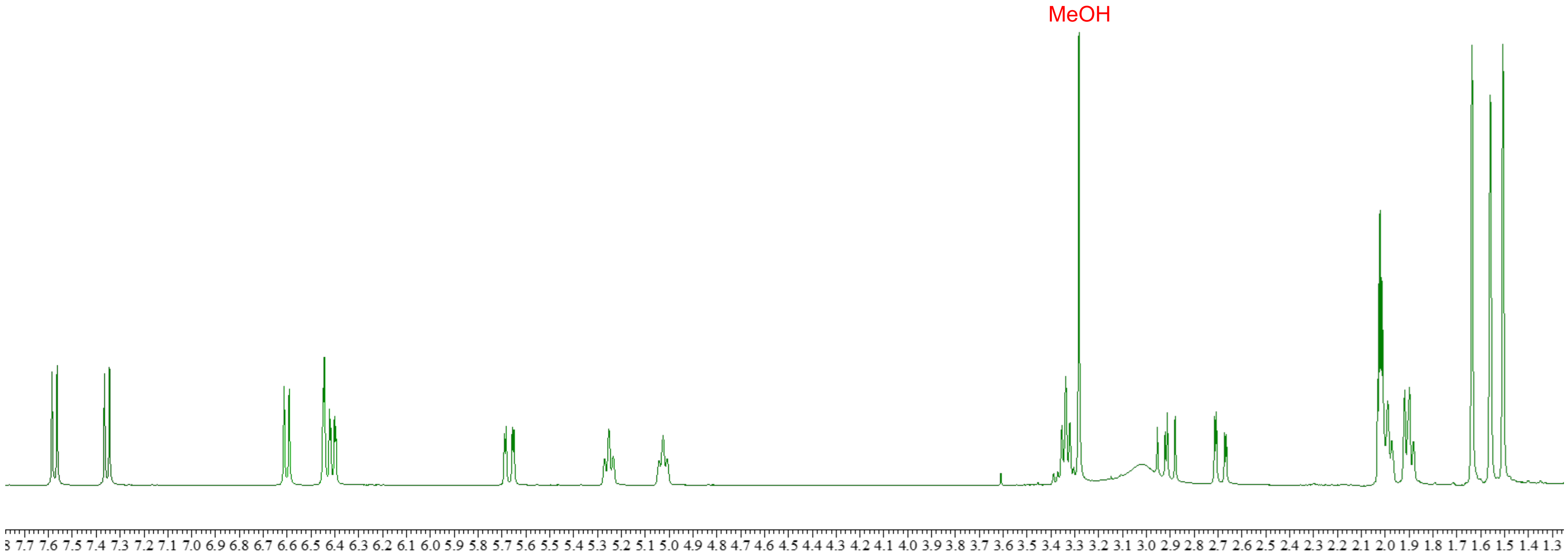

Figure S1. ^{1}H NMR spectrum of malheuran A (400 MHz, acetone-d_6).

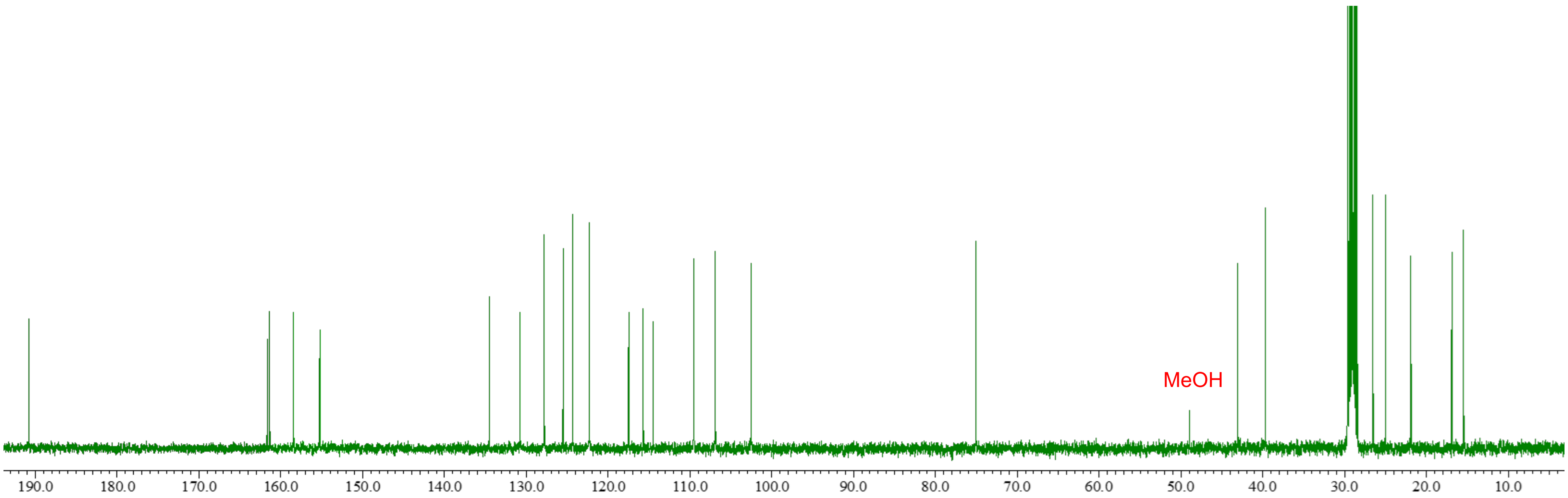

Figure S2. ^{13}C NMR spectrum of malheuran A (101 MHz, acetone-d_6)

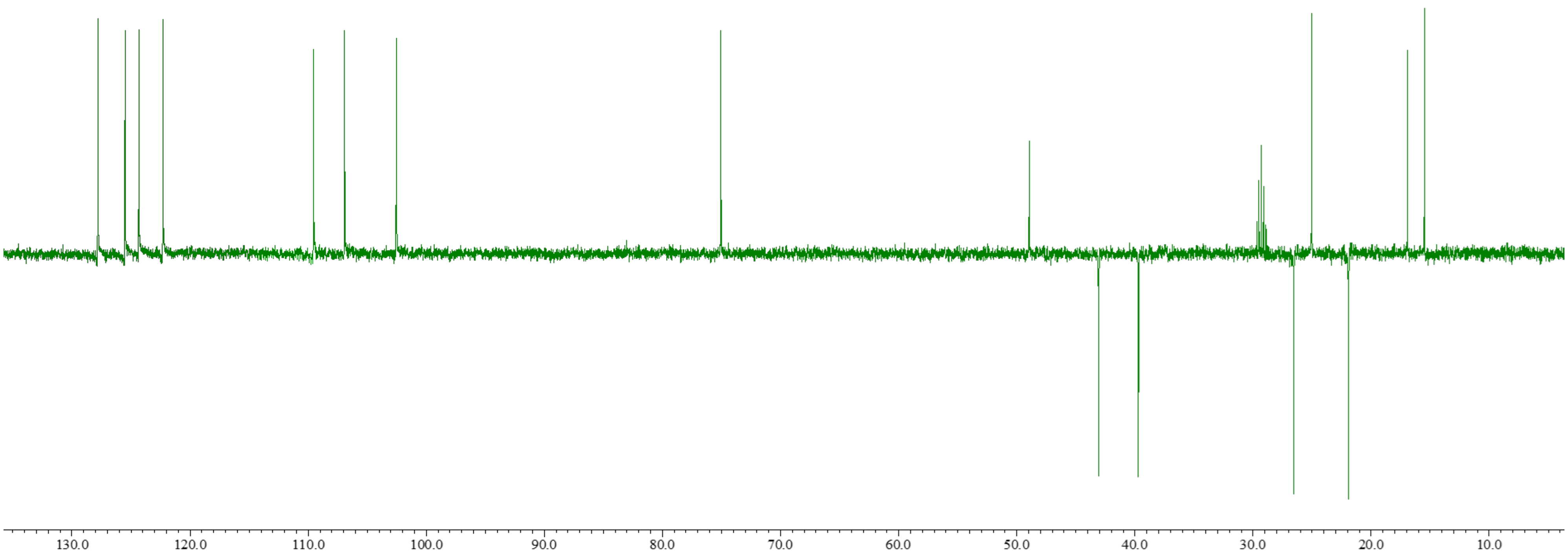

Figure S3. DEPT135 spectrum of malheuran A (101 MHz, acetone-d_6).

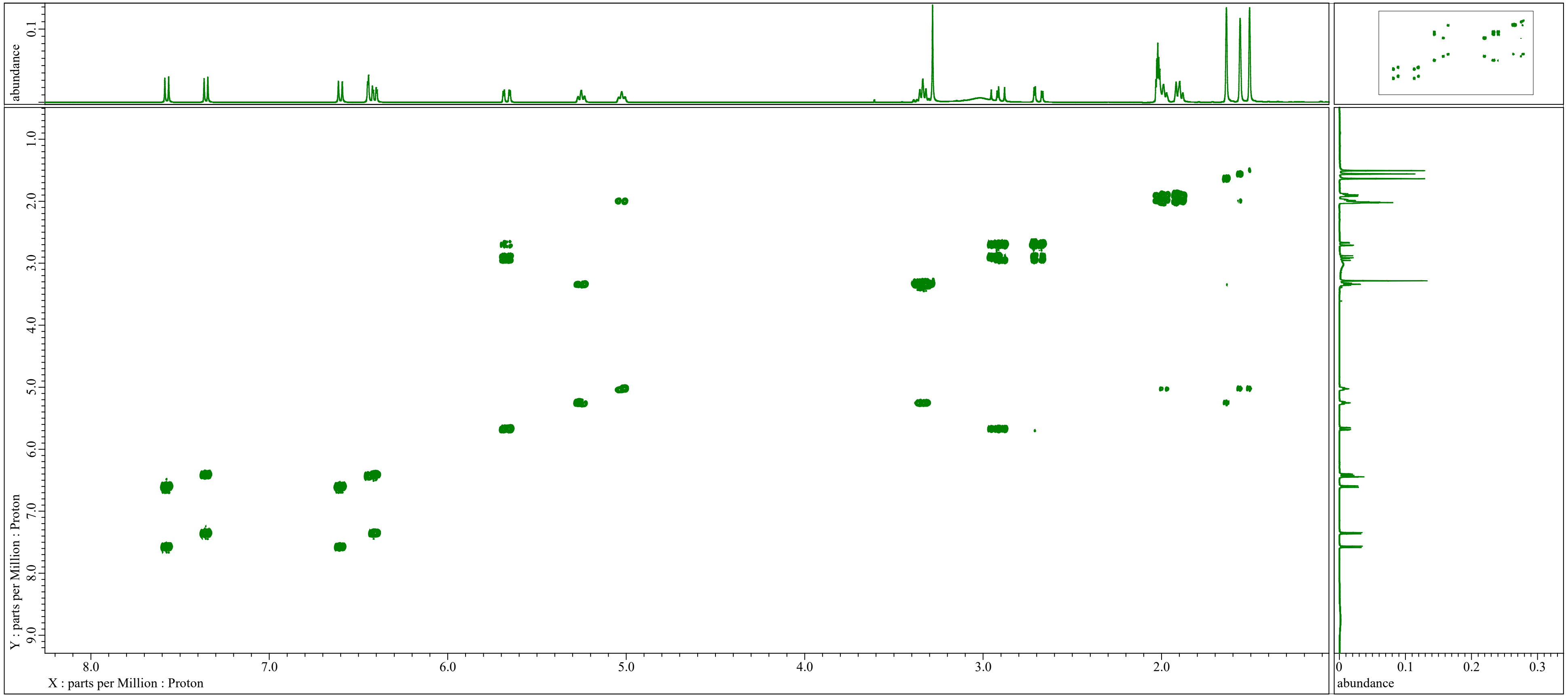

Figure S4. COSY spectrum of malheuran A (400 MHz, acetone-d_6).

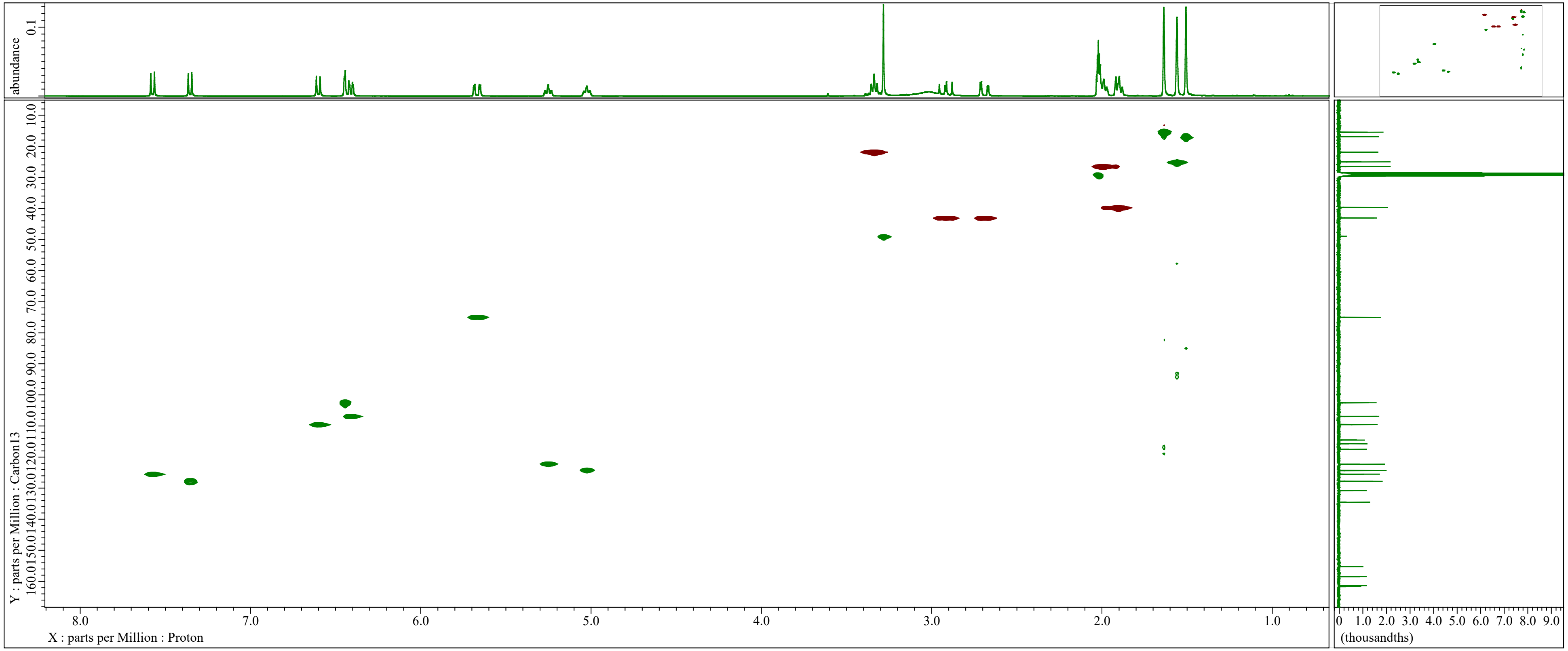

Figure S5. HSQC spectrum of malheuran A (400 MHz, acetone-d_6).

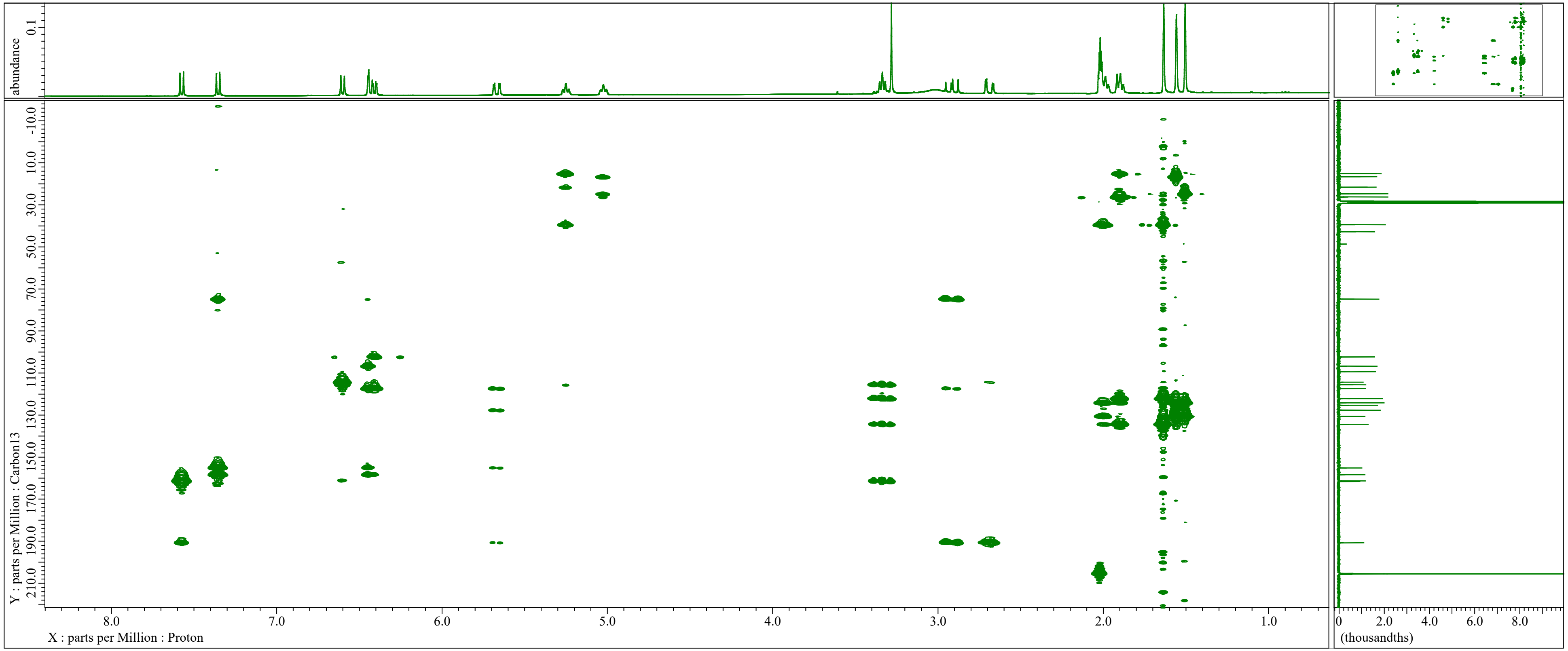

Figure S6. HMBC spectrum of malheuran A (400 MHz, acetone-d_6).

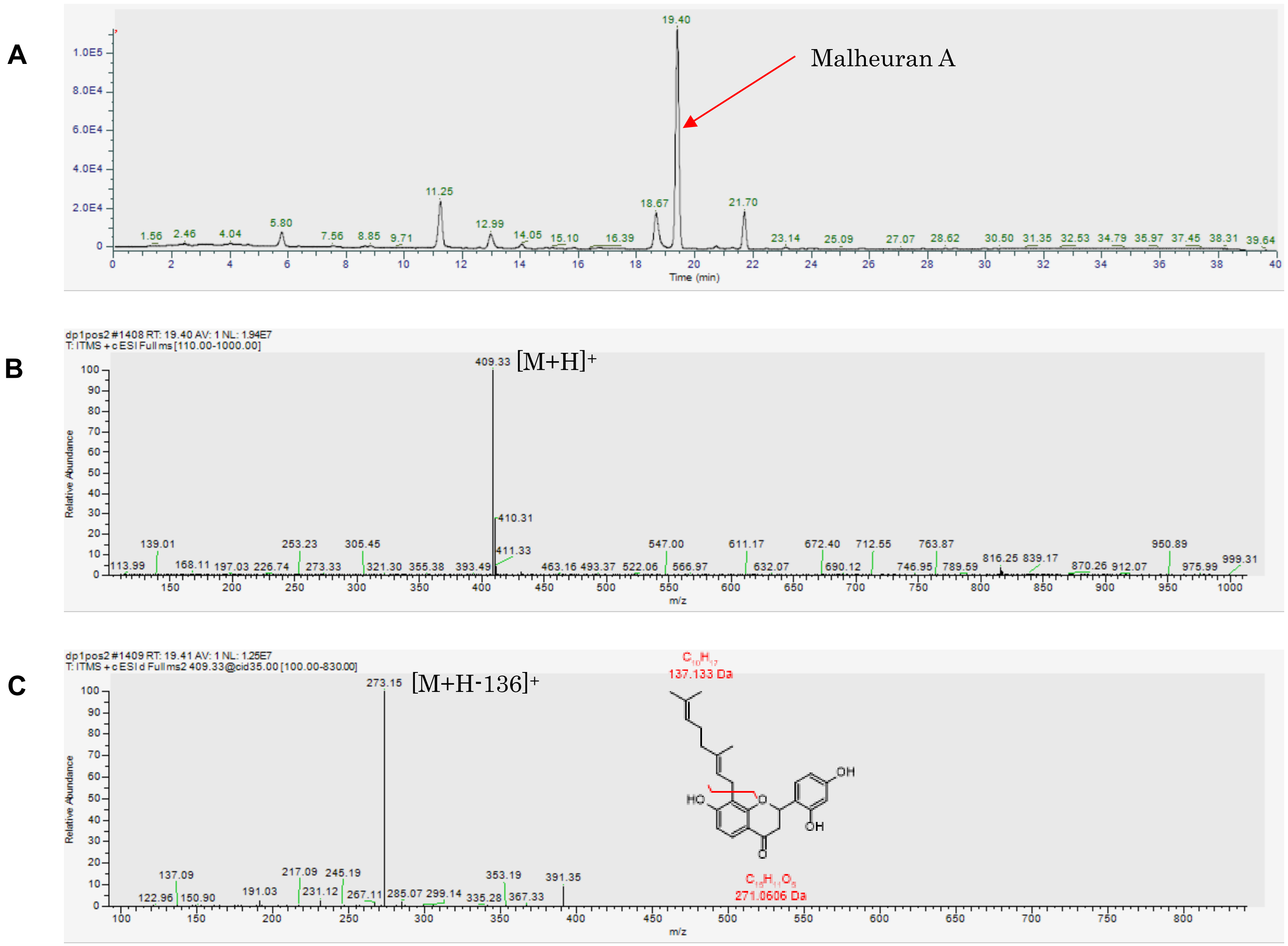

Figure S7. LC-MS analysis of malheuran A from CD+H_2O_2+$MgCl_2$+MeJA elicited extract in positive ion mode. (**A**) HPLC chromatogram; (**B**) MS ion chromatogram; (**C**) MS^2 ion chromatogram.

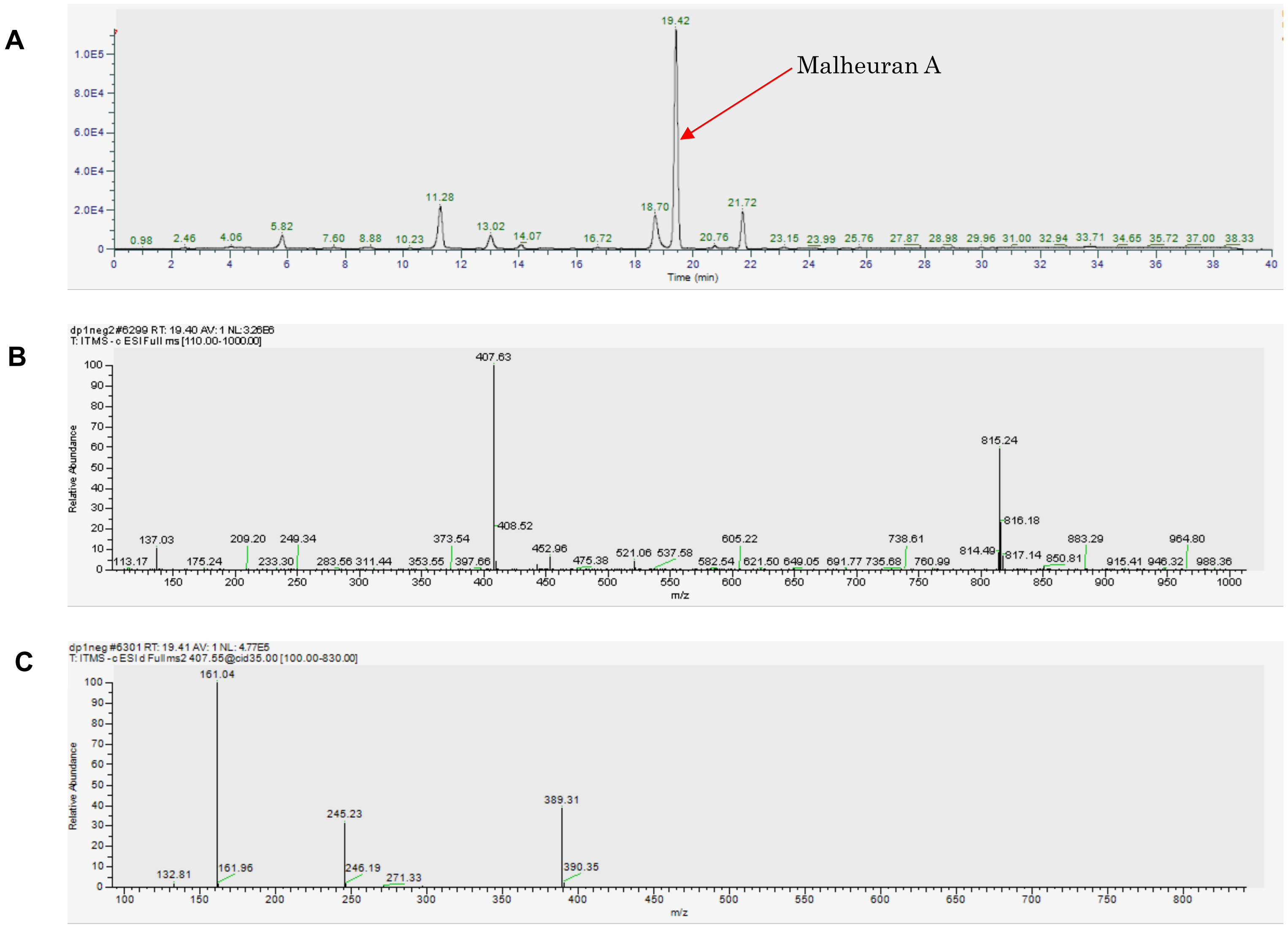

Figure S8. LC-MS analysis of malheuran A from CD+H_2O_2+$MgCl_2$+MeJA elicited extract in negative ion mode. (**A**) HPLC chromatogram; (**B**) MS ion chromatogram; (**C**) MS^2 ion chromatogram.
